# Post-Laminectomy Kyphosis in Achondroplasia Patients: To Concurrently Fuse or Not

**DOI:** 10.7759/cureus.7966

**Published:** 2020-05-05

**Authors:** David R Hallan, Oliver D Mrowczynski, Sarah McNutt, Elias Rizk

**Affiliations:** 1 Neurosurgery, Penn State Milton S. Hershey Medical Center, Hershey, USA

**Keywords:** laminectomy, kyphosis, achondroplasia, review

## Abstract

The purpose of this review is to look at the incidence of post-laminectomy kyphosis in achondroplasia patients and to determine whether skeletal maturity and the number and location of laminectomies predict kyphosis in this patient population. Our review of the literature included all articles from MEDLINE/PubMed and Ovid from inception to 2019. After removing duplicates and checking for relevancy, the final number of articles yielded was eight. The results of this review summarize the incidence of post-laminectomy kyphosis in achondroplasia patients. In conclusion, we suggest fusion be considered in conjunction with multilevel laminectomies due to a high incidence of kyphosis with a need for stabilization in the pediatric achondroplastic patient population.

## Introduction and background

Achondroplasia is the most common form of human skeletal dysplasia, arising from failure of endochondral ossification and longitudinal bone growth, with an estimated incidence of one in 28,000 [1,2]. Achondroplasia is due to an autosomal dominant activating mutation with 100% penetrance in the fibroblast growth factor receptor 3 (FGFR3) gene [1-3]. The constitutive activation of FGFR3 inhibits chondrocyte proliferation resulting in impaired endochondral ossification. A heterozygous mutation is most common, as a homozygous mutation is often lethal.

Spinal stenosis

Spinal canal narrowing is common in patients with achondroplasia due to anatomical deformities including short stature with a normal spine length, 30% to 40% thicker pedicles, and progressively narrowing interpedicular spacing caudally. Other clinical findings include increased angle between the lumbar spine and sacrum, thickened lamina, intervertebral disc widening, and scalloped posterior vertebra. These deformities increase the incidence of spinal cord compression and cauda equina [2-8]. Additionally, kyphosis may be present at the thoracolumbar junction, leading to vertebral bodies wedging and forming a “bullet-shaped” vertebra cranially as the kyphosis progresses [1]. Spinal stenosis generally worsens with age as the ligamentum flavum hypertrophies, epidural fat decreases, and degenerative spondylosis develops [2,8]. In many cases, the neurological sequelae caused by these anatomic differences have necessitated surgical treatment, with laminectomy being the most common [2]. 

In 1988, Hall reported that 20% to 30% of achondroplastic patients have spinal stenosis symptoms at some point in their lifetime, with almost 10% requiring intervention [2,9]. In 1998, Hunter et al. reported that 42.1% of achondroplastic patients have spinal stenosis and 24.1% require surgery during their life [2,10]. In 2006, Ain reported that 37% to 89% of achondroplastic patients have symptomatic spinal stenosis, but only 10% to 25% will require surgical treatment [8,11]. And in 2010, Baca et al. reported that by adulthood, 20% to 30% of achondroplastic patients will have neurogenic symptoms (including paraplegia, bowel/bladder dysfunction, or walking intolerance) with 10% of them requiring surgical intervention [4].

Spinal stenosis in achondroplasia patients usually develops in early adolescence [2]. However, symptomatic spinal stenosis is uncommon until the third or fourth decade of life. Though it can occur in patients of any skeletal maturity, the average age on admission is 31 years [8,11-14]. When spinal stenosis does become symptomatic in skeletally immature patients, delaying intervention by surgical decompression can result in permanent dysfunction [8]. Surgical decompression often includes multilevel foraminotomies with laminectomies and undercutting of the facets [8,11,14].

Schkrohowsky et al. in 2007 determined that skeletally immature achondroplasia patients who required surgery when compared to those that did not present with a decreased T12-L5 interpedicular distance from -8% to -19%. These patients also had a significantly increased thoracolumbar kyphosis (TLK) angle from 24.2 to 14.1 degrees, 100% had neurogenic claudication symptoms, and 77% presented with bladder incontinence [13]. The exact timing, procedure, and indications for surgery in achondroplasia patients with spinal stenosis are not well established but indications for surgical decompression may include gait disturbances, paraplegia, weakness, pain, numbness, cramps, incontinence, and spasms [7,13,14].

Why we are looking at post-laminectomy kyphosis

Destabilization of the vertebral column is a known risk of extensive surgical decompression [8]. However, despite approximately 25% of achondroplastic patients requiring surgical intervention due to spinal stenosis, the risk of post-laminectomy kyphosis is not well established [2,8]. This risk is important to consider because post-laminectomy kyphosis is a difficult condition to manage and may require a second surgery, making prevention preferable [2]. Multiple devastating clinical sequelae may occur from post-laminectomy kyphosis, including increased risk of neurologic injury and deformity. Currently, information regarding surgical management of post-laminectomy kyphosis in children is lacking [2].

The importance of monitoring patients for post-laminectomy kyphosis has been repeatedly emphasized over the past 40 years [15,16]. However, most current reported evidence for post-laminectomy kyphosis is not specific for achondroplastic patients but rather for patients below 18 years who have had a laminectomy for treatment of spinal tumors [2]. The incidence of post-laminectomy kyphosis in adults is rare. The incidence of post-laminectomy kyphosis in children and adolescents with achondroplasia remains unknown [8].

Past recommendations to prevent kyphosis include prophylactic bracing and laminectomy with a concurrent anterior and posterior spinal fusion of the affected area [2,16,17]. Nevertheless, bracing has not been shown to be effective [2,12].

Here, we present a review of the literature on post-laminectomy kyphosis in achondroplasia patients and summarize the available reported cases to underline the usefulness, effectiveness, and complications of this treatment method.

## Review

Materials and methods

Study selection: Our review of the literature included all articles from MEDLINE/PubMed and Ovid from inception to 2020. Search terms included combinations of achondroplasia and kyphosis, achondroplasia and laminectomy, achondroplasia and laminectomy and kyphosis, post-laminectomy and achondroplasia, and post-laminectomy kyphosis, which produced 123 articles. Irrelevant and duplicate articles were removed. The references of retrieved articles were searched for further relevant studies. The final number of studies included those with a patient population of achondroplasia patients who had laminectomies (Table 1). Chi-square analysis and a Cochran-Mantel-Haenszel test for the general association to adjust for stratified results were performed (Table 2).

**Table 1:** Characteristics of patients with achondroplasia who underwent decompressive surgery for symptomatic spinal stenosis

Author and year of publication	Trial design	No. of patients	% Male (no.)	% Female (no.)	Mean age at surgery (years)	Type of intervention	Pre-surgery kyphosis angle	Post-surgery kyphosis angle	Follow-up range (years)	Laminectomy only + no revision needed (no.)	Laminectomy with concurrent fusion + no revision needed (no.)	Laminectomy alone + revision needed (no.)	Laminectomy with concurrent fusion + revision needed (no.)
Pyeritz 1987 [12]	Retrospective Review	22	64 (14)	36 (8)	32.8	Multilevel laminectomy (average 5.7 segments) without concurrent fusion	Unmeasured	Unmeasured. Worsened in three patients, two requiring fusion	8	20	0	2	0
Streeten 1988 [11]	Retrospective Review	20	45 (9)	55 (11)	33.7	Multilevel laminectomy (average 10.7 segments) without concurrent fusion	Unmeasured	Unmeasured	0.65	13	0	2	0
Hahn 1989 [18]	Case Report	1	-	-	0.58 (7 months)	Laminectomy T11-L1 without concurrent fusion	Unmeasured, but present in 100%.	Unmeasured	3	1	0	0	0
Ain 2006 [8]	Retrospective Review	10	60 (6)	40 (4)	9.2	Multilevel thoracolumbar laminectomies, involving 5–8 levels, without concurrent fusion	Mean of 31 degrees	Mean of 94 degrees	1.1	0	0	10	0
Sciubba 2007 [17]	Retrospective Review	44	57 (25)	43 (19)	12.7	Laminectomy with and without concurrent fusion (17 without)	Unmeasured, but present in 50%	Unmeasured. Five of 17 who weren't fused developed "progressive deformity"	2.8	6	32	11	0
Agabegi 2008 [2]	Case Report	1	0	100 (1)	12	T12-L5 laminectomy without concurrent fusion	Unmeasured	Mean of 105 degrees	4	0	0	1	0
Baca 2010 [4]	Retrospective Review	18	61 (11)	39 (7)	10.6	Five- to 8-level thoracolumbar laminectomy, half with fusion (if Risser grade <5) and half without fusion	Less than 50 degrees in 100%	Nine patients developed kyphosis >70 degrees	6	2	7	7	2
Vleggeert-Lankamp 2012 [7]	Retrospective Review	20	50 (10)	50 (10)	51.2	Multilevel laminectomy (average 2.1 segments) without concurrent fusion	Mean of 22.6 degrees	No increase	3.2	20	0	0	0

**Table 2:** Chi-square analysis of achondroplastic patients under the age of 18 undergoing surgery Each cell includes the observed cell total, (the expected cell totals), and [the chi-square statistic]. Chi-square statistic is 42.2131, with a p-value of <0.00001.

	Laminectomy	Laminectomy with concurrent fusion	Total
No revision needed	9 (23.09) [8.60]	39 (24.91) [7.97]	48
Revision needed	29 (14.91) [13.31]	2 (16.09) [12.34]	31
Total	38	41	79

Results

In 1987, Pyeritz et al. published a retrospective study on the long-term effects of thoracolumbosacral laminectomy in 22 achondroplasia patients. They reviewed the clinical history of patients who had at least one thoracolumbosacral laminectomy performed before 1981. Follow-up after the first laminectomy averaged eight years. Of those 22 patients ages 13.5-61.5, with average age 32.8 years, kyphosis occurred in three patients, with two patients requiring fusion for post-laminectomy kyphosis [8,12].

In 1988, Streeten et al. published a retrospective study looking at the post-operative course of 20 achondroplasia patients who received extended laminectomy for spinal stenosis. The average age at surgery was 33.7 years, with a range of age 10.5-54.8. The average length of the laminectomy segment was 10.7 segments. With an average follow-up of 9.8 months for 16 of 20 patients with whom follow-up was possible, the researchers did not make a note of any kyphosis post-operatively [11].

In 1989, Hahn et al. published a case report of a seven-month-old achondroplasia patient who developed paraplegia secondary to thoracolumbar spinal cord compression at T12-L1. A pre-operative X-ray showed scoliosis and kyphosis. The patient received a decompressive laminectomy from T11 to L1. The child recovered from his or her paraplegia, and after being followed for three years no kyphosis was noted [18].

In 2006, Ain et al. published a retrospective study looking at post-laminectomy kyphosis in 10 consecutive achondroplasia patients with an average age of 9.2. All 10 patients had wide-level laminectomy of five to eight levels for symptomatic spinal stenosis without fusion. Post-laminectomy kyphosis developed in all 10 patients ranging from 78 to 135 degrees with a mean of 94 degrees. The mean increase in kyphosis from pre- to post-op was 63 degrees. Kyphosis occurred regardless of early mobilization and, for two patients, bracing. All 10 patients received fusion an average of 13.2 months after an initial laminectomy to stabilize the kyphosis. After revision, 80% of the patient’s pain improved. Ain et al. concluded that “concurrent spinal fusion is indicated in skeletally immature achondroplastic patients who undergo thoracolumbar laminectomies of at least 5 levels” [8].

In 2007, Sciubba et al. published a retrospective study looking at post-laminectomy kyphosis, deformity, and complications in 44 pediatric patients with achondroplasia over a nine-year time period. Of the 44 patients, 22 of them had preexisting kyphosis prior to laminectomy, but only five developed progressive kyphosis after decompression without concurrent fusion. The average age at operation was 12.7 ± 4.4 years. Forty-nine primary surgeries were performed: 32 fusions and 17 laminectomies. Eleven revisions with fusion were needed. Ten out of 11 revisions were necessary due to progressive deformity or junctional stenosis. Sciubba et al.’s study shows there may be a substantial clinical benefit to spinal decompression of pediatric achondroplastic patients when performed safely at any level. They further demonstrate that avoidance of progressive postoperative kyphosis can be achieved with fusion [17].

In 2008, Agabegi et al. published a case report of a 12-year-old girl with achondroplasia who presented with low back pain, bilateral leg pain, and urinary incontinence of one-year duration. Pre-operative X-rays showed lordosis of her entire thoracolumbar spine. A T12-L5 laminectomy was performed for decompression without complication. Over the next four years of follow-up, the patient developed a TLK progressing to 105 degrees, despite being treated with a brace when first noticed. Fusion was then performed for stabilization [2].

In 2010, Baca et al. published a retrospective review of 18 patients under the age of 18 with achondroplasia who underwent spinal decompression. The mean follow-up time was 72 ± 27.6 months. On pre-operative evaluation, all 18 patients had kyphosis less than 50 degrees with normal sagittal alignment of the thoracolumbar junction. All patients had laminectomies of at least five levels, up to eight levels. Nine of the patients underwent instrumentation at the time of laminectomy, and nine patients did not. Revision surgeries were required by nine patients, two of which had an initial fusion, and seven who did not. All nine developed a kyphosis greater than 70 degrees. Eight had progressive symptomatology, and one underwent revision surgery for kyphosis without symptomatology. Researchers concluded that patients had 3.5 times increased chance of revision surgery if they did not have instrumentation placed initially. They state that although laminectomy without fusion can be safe in pediatric (skeletally immature) achondroplasia patients in some cases, in most, decompression without fusion leads to worsening kyphosis and neurogenic symptomatology [4].

In 2012, Vleggeert-Lankamp and Peul published a retrospective review of 20 patients with achondroplasia who underwent spinal decompression. The average age at the time of surgery was 51.2 ± 12.7 years, with age from 21 to 67 years old. Pre-operatively, a thoracolumbar kyphotic angle was measured using fluoroscopy and alternative Cobb method, producing a mean angle of 22.6 ± 12.5 degrees. No post-operative increase in angle was reported. Indeed, the two patients with a “significant kyphotic angle” who had laminectomy without instrumentation had no deterioration in clinical symptoms after a mean of 38 months' follow-up after surgery. However, the authors conclude that in achondroplastic patients with thoracic spinal cord compression, evaluation of the pre- and post-operative thoracolumbar angle, as well as angle progression, is still important [7].

To determine the relationship between requiring revision surgery and the type of initial surgery (laminectomy or fusion), we performed a Chi-square analysis and a Cochran-Mantel-Haenszel test for the general association to adjust for stratified results (Table 2).

The study was the stratification factor for the test. Only pediatric cases were included from the data provided in Table 1, and only revisions required for kyphosis were recorded. The test for general association shows a significant association between the two variables (p < 0.00001). The adjusted odds ratio shows that an achondroplastic pediatric patient with laminectomy has 44.57 times the odds (is more likely) to have revision versus those with laminectomy plus fusion. Even at the lower limit of 95% confidence, this is still 6.81 times the odds. Therefore, we determined there is a statistically significant relationship between having an initial laminectomy and requiring a revision and/or fusion in the pediatric (under 18 years of age) achondroplastic population. This same type of analysis could not be created for non-pediatric cases, as no adult patients received a fusion at the time of laminectomy. Of the 57 adult patients who underwent laminectomy, only four of them (7%) required a revision and fusion. In both pediatric and adult cases, there is insufficient data to determine whether the extent and levels of laminectomy are predictive of needing concurrent fusion.

Discussion

Post-laminectomy kyphosis has a higher incidence in pediatric patients with achondroplasia than the general population. This incidence is likely due to a number of factors inherent to achondroplasia, including increased laxity of ligaments, enlarged head relative to the body, decreased muscle tone of trunk extensor muscles, and increased degree of pre-operative kyphosis. Other factors include continued growth of the axial skeleton with incomplete ossification and the level and extent of laminectomy needed to be performed due to higher incidences of spinal stenosis.

In order to best understand how a laminectomy may influence kyphosis, it is first necessary to understand the natural history of kyphosis in the achondroplastic patient. First described by Wheeldon in 1920, TLK in achondroplasia is usually noticeable either at or within the first few months of birth. The TLK worsens as the achondroplastic dwarf becomes able to sit up but resolves spontaneously in roughly 70% of cases when able to stand. In the remaining 30% or so of cases where the TLK does not resolve, it can worsen, hypothesized to be due to compensation for lumbosacral hyperlordosis, abnormal development of vertebral metameres, or the limited anterior longitudinal growth at the epiphyseal plates [15,19]. Sciubba et al. report that TLK is present in approximately 11% of achondroplastic patients at 10 years old, and approximately 30% or greater of achondroplastic patients at age 30 years or older [17].

In 2012, Engberts et al. assessed the existing evidence of the prevalence of TLK in achondroplasia. They hoped to clarify the degree of kyphosis constituting TLK, what measurement method to use, and how this information could be used to aid in treatment selection. Their literature search resulted in seven studies with a reported prevalence of TLK in the achondroplasia patient of 50% to 100%. However, the definition of TLK and method of measurement were not included in the reviewed studies. In addition, all studies had a limited sample size, making an estimate of TLK prevalence difficult. They concluded that there is very little information available on the prevalence of TLK in achondroplasia, and the association between TLK and age cannot be inferred [6].

Although any kyphosis at the thoracolumbar junction can be thought of as pathological, what angle constitutes the need for intervention, and at what age? Lonstein reports that surgery is necessary for any acute angular TLK (as orthotic treatment is ineffective), any progressive kyphosis, and any kyphotic deformity over 60-70 degrees in children, but does not give numerical evidence to support these recommendations [16]. Occasionally in achondroplasia, kyphosis alone can result in spinal cord compression and neurological symptoms, including mild paraparesis to complete paralysis. A surgical correction would obviously be indicated in these cases.

Laminectomies are frequently performed in non-achondroplastic patients, yet deformity following laminectomy in these cases is very rare. Laminectomy is also often performed in children diagnosed with spinal cord tumors, and spinal deformity is common post-operatively [20]. This was not originally believed to be the case, as a large number of children did not survive this condition until recent aggressive treatments with laminectomy, tumor excision, radiotherapy, and chemotherapy became standard. However, with increased survival came increased knowledge about deformity post-laminectomy. Incidence of kyphosis in children treated with laminectomy for tumors has been shown to be 33% to 78%. In 1959, Haft et al. reported that of 30 children who underwent laminectomy for spinal cord tumor, 17 survived and 10 went on to develop spinal deformity: an incidence of 33% [21]. In 1965, Tachdjian and Matson reported that of 115 children, 46 of them (40%) developed spinal deformity [22]. In 1977, Lonstein reported that of 32 children, 16 of them (50%) developed spinal deformity. Overall, following laminectomy, kyphosis was the most common deformity seen occurring most commonly at the site of laminectomy in the cervicothoracic and thoracic areas of the spine, averaging 78 degrees [20].

Factors that lead to the development of post-laminectomy kyphosis

In general, a higher incidence of spinal deformity post-laminectomy has been reported in younger patients than in adult patients [2,4,8]. This general statement extends itself to the achondroplastic skeleton as well (Table 1). In addition, a greater number of levels operated on, as well as laminectomies in the lower thoracic or lumbar spine, correlate with a higher incidence of kyphosis. The loss of bone and soft tissue naturally leads to destabilization [20]. The loss of posterior ligamentous tension bands reduce posterior stability, increases anterior stressors on the incompletely ossified cartilaginous apophysis, and ultimately causes a vertebral body wedge deformity [2,17]. Preserving maximum tension while relieving compression may be prudent to decrease the incidence of kyphosis. In the non-achondroplastic patient, age is hypothesized to contribute to a higher incidence of post-laminectomy kyphosis because as the axial skeleton continues to grow, the vertebral bodies are incompletely ossified and the intervertebral ligaments have greater viscoelasticity [2,8]. The same holds true for achondroplastic patients, with the added risk factors of an enlarged head relative to the body, increased laxity of ligaments, and decreased muscle tone of trunk extensor muscles [2,15]. Pre-operative kyphosis is another likely pre-disposing risk factor to post-operative kyphosis and concurrent fusion in all patients with achondroplasia undergoing decompression who have pre-operative TLK has been recommended [8,16,17]. In fact, if the pre-operative kyphosis exceeds 50 degrees, then anterior-posterior fixation may be required [17].

Limitations

Several limitations are acknowledged by the authors in the present study. One limitation is that the reported outcomes of significant side effects are derived from different institutions. This may lead to subsequent differences in reporting when a patient has kyphosis, pain, paralysis, etc. The inconsistency between institutions may also include but is not limited to how the procedures are performed, methods of post-operative care, and thoroughness of follow-up. Another limitation noted by the authors is due to the studies included in this review are in the vast majority retrospective, results have not been looked at through a randomized controlled trial. Thus some publication bias may exist. The search was limited to English language publications. Finally, the aggregate design of this study makes accounting for all variables difficult.

## Conclusions

Using published literature as a data source, we demonstrated that a significant relationship between initial laminectomy in this population and requiring an additional fusion exists. Concomitant stabilization with fusion should be considered when performing wide-level laminectomies in pediatric achondroplasia patients. The same may not hold true for adult achondroplastic patients. Furthermore, it was unable to be determined whether the levels of laminectomy and the number of segments in both pediatric and adult patients are predictive of kyphosis development or subsequent need for a fusion. The authors acknowledge that the literature on this subject is sparse, and accounting for other variables is difficult. Ultimately, we recommend that this be looked at closer in the registry format.
